# Empyema

**Published:** 1907-12-28

**Authors:** 


### Points in Surgery.

### EMPYEMA.

Empyema is a condition which nearly always comes under the notice of the surgeon secondarily; that is to say that he is called in to operate upon an empyema whose presence has already been diagnosed with certainty. But this fact does not excuse the surgeon for ignorance of the signs and symptoms of the condition. Empyema is a common sequela of lobar pneumonia, and in such cases the pus is generally found to contain a pure culture of the pneumococcus. In a certain number of cases, the pus is due to one of the ordinary pyogenic micro-organisms, such as the streptococcus or staphylococcus, and except in the case of a compound fracture of the rib involving the pleural sac the pathology of these cases is somewhat obscure. It must be supposed that the infection is a hæmic one. In rare cases both pneumococci and septic micro-organisms can be cultivated from the pus of the same empyema.

### Diagnosis.

It is of great importance to diagnose the condition early, because immediate surgical treatment is indicated. It is an unfortunate fact, but a true one, that many cases of empyema are not even suspected, far less diagnosed, as early as they should be, even in the wards of a hospital where the patient is under continual observation, and any change in his condition is noted from day to day. As a general rule it may be said that any patient convalescing from lobar pneumonia should be carefully watched on the chance of his developing an empyema, even when he is apparently quite convalescent. The onset of a rigor, with a rise of temperature, should put one on one's guard; and should these occur the chest should at once be examined for the physical signs of a pleural effusion. The details of such an examination do not of course, lie strictly within the province of the surgeon; but there are certain gross signs with which he should be familiar. They are (i.) displacement of the apex-beat of the heart; if the empyema is left-sided the apex is displaced towards the middle line, while if it is right-sided the apex is displaced in a typical case right out towards the mid-axillary line; (ii.) the presence of a small or large area of the thoracic wall which is dull on percussion; (iii.) breath sounds are absent over the dull area, but ægophony is a fairly constant phenomenon; (iv.) at the upper limit of the dull area there may be tubular breathing. If the empyema has existed for some time before its presence is discovered there may be œdema of the chest wall, and the patient may present an emaciated appearance, with a hectic flush.

The presence of these signs is quite sufficient evidence to show that there is some free fluid in the pleural cavity. Whether the fluid is purulent or sero-fibrinous it is almost impossible to say without aspirating the thorax. At any rate, none of the clinical tests which have been advocated for differentiation between the two can be said to be convincing. In any case it is wise to remove some of the fluid with an exploring syringe, because if it turns out to be sero-fibrinous the remainder of the effusion should be removed with an aspirating-bottle, while, if it is purulent, more radical surgical treatment is called for as soon as possible.

### The Importance of Early Operation.

Early operation is essential because an empyema that is unrelieved is a very dangerous possession. It is said that small pneumococcal empyemata are occasionally absorbed spontaneously; this termination, if it ever really happens, must be excessively rare. The pus may be spontaneously evacuated by making its way into the lung and so being discharged through the bronchi. But, as might be expected, this generally leads to a fatal septic broncho-pneumonia or to gangrene of the lung itself. In other cases the pus may make its way to the surface of its own accord: the name of empyema necessitatis has been given to this state of affairs. The point where the pus comes to the surface is generally situated in the fifth costal interspace, just external to the margin of the pectoralis major. Or again an untreated empyema may cause death by mechanical interference with the heart's action owing to the pressure of the contained fluid, or by the absorption of toxins if the process be an acute one. If the infection causing the empyema is not a very acute one none of these things may occur, but the inflamed pleural wall may become chronically thickened and bound down by adhesions to the thoracic wall, so that even after evacuation of the pus the lung is unable to expand, and in this case a cure can only be effected by taking such measures as will allow the thoracic wall to fall in on the collapsed lung, a result which leads to much deformity.

It will therefore be seen that the moment the clinical signs point to the presence of an effusion into the pleural sac, it is the duty of the surgeon to confirm the diagnosis by exploring the pleural cavity without loss of time. For this purpose the chest wall over the dull area is made thoroughly aseptic. This is a most important point, since, if the asepsis is imperfect there is a danger of introducing pyogenic micro-organisms into an effusion which was either a simple serous effusion or one which contained only pneumococci. The syringe used should be a special one, with a barrel large enough to hold one or two ounces of fluid, and a needle sufficiently long to go through the thoracic parietes and pleura, and having a fairly wide bore, so that it does not get plugged up with flakes of lymph. If the empyema is generalised the needle may be inserted in the sixth intercostal space in the mid-axillary line; but, of course, if it be localised it must be put in at the middle of the dull area, wherever that may be. The actual introduction of the needle is not difficult. The parts are steadied with the left hand, while the needle is introduced along the upper margin of the lower of the two ribs bounding the space.

*(To be continued.)*

